# A field test of computer-vision-based gaze estimation in psychology

**DOI:** 10.3758/s13428-023-02125-1

**Published:** 2023-04-26

**Authors:** Niilo V. Valtakari, Roy S. Hessels, Diederick C. Niehorster, Charlotte Viktorsson, Pär Nyström, Terje Falck-Ytter, Chantal Kemner, Ignace T. C. Hooge

**Affiliations:** 1 Experimental Psychology, Helmholtz Institute, Utrecht University, Heidelberglaan 1, 3584 CS Utrecht, the Netherlands; 2 Lund University Humanities Lab, Lund University, Lund, Sweden; 3 Department of Psychology, Lund University, Lund, Sweden; 4 Development and Neurodiversity Lab, Department of Psychology, Uppsala University, Uppsala, Sweden; 5 Uppsala Child and Baby Lab, Department of Psychology, Uppsala University, Uppsala, Sweden; 6 Karolinska Institutet Center of Neurodevelopmental Disorders (KIND), Department of Women’s and Children’s Health, Karolinska Institutet, Stockholm, Sweden

**Keywords:** Eye tracking, Gaze estimation, Computer vision, Data quality

## Abstract

Computer-vision-based gaze estimation refers to techniques that estimate gaze direction directly from video recordings of the eyes or face without the need for an eye tracker. Although many such methods exist, their validation is often found in the technical literature (e.g., computer science conference papers). We aimed to (1) identify which computer-vision-based gaze estimation methods are usable by the average researcher in fields such as psychology or education, and (2) evaluate these methods. We searched for methods that do not require calibration and have clear documentation. Two toolkits, OpenFace and OpenGaze, were found to fulfill these criteria. First, we present an experiment where adult participants fixated on nine stimulus points on a computer screen. We filmed their face with a camera and processed the recorded videos with OpenFace and OpenGaze. We conclude that OpenGaze is accurate and precise enough to be used in screen-based experiments with stimuli separated by at least 11 degrees of gaze angle. OpenFace was not sufficiently accurate for such situations but can potentially be used in sparser environments. We then examined whether OpenFace could be used with horizontally separated stimuli in a sparse environment with infant participants. We compared dwell measures based on OpenFace estimates to the same measures based on manual coding. We conclude that OpenFace gaze estimates may potentially be used with measures such as relative total dwell time to sparse, horizontally separated areas of interest, but should not be used to draw conclusions about measures such as dwell duration.

## Introduction

Eye movements are integral to human behavior, and gaze direction can be used to infer different aspects of cognitive function. As such, studies have been conducted on how infants model and navigate the world and categorize objects (Franchak et al., 2011; Gredebäck et al., 2009; Johnson et al., 2003; Oakes, 2012), how adults acquire information in specific situations (Ballard et al., 1995; Hayhoe, 2004; Hayhoe & Ballard, 2005), and how people interact with each other face-to-face (Hessels, 2020). In sum, there is an abundance of research investigating the role of gaze in different aspects of human behavior (see, e.g., Land & Tatler, 2009; Duchowski, 2017).

Current gaze research typically employs eye trackers using the pupil minus corneal reflection (p-CR) technique (see, e.g., Hooge et al., 2016, for an explanation). Although eye trackers can provide an accurate and reliable estimate of gaze direction, they need to be placed either in front of or on the head of the participant, usually need to be calibrated for each participant, and often only allow for limited head and upper body movement (see Holmqvist et al., 2011; Holmqvist & Andersson, 2017, for further discussion on the different types of p-CR eye trackers and setups). An alternative technique to estimate gaze direction, *computer-vision-based gaze estimation*, shows promise in mitigating these limitations.

Computer-vision-based gaze estimation refers to methods that can be used to estimate gaze direction solely from video recordings or images of the eyes and/or face without the need for an eye tracker (see Hansen & Ji, 2009, for detailed discussion on different gaze estimation methods). Computer-vision-based gaze estimation typically employs either a model- or appearance-based approach (Zhang et al., 2017). Model-based approaches first detect the locations of specific eye region landmarks and then fit those into a three-dimensional (3D) model of an eyeball to estimate gaze direction (e.g., Baltrušaitis et al., 2018; Park et al., 2018). Model-based approaches may also further employ machine learning to improve detection performance. Appearance-based approaches, on the other hand, estimate gaze direction directly from how the eyes and/or face appear in a 2D video image using machine learning, without any underlying model of the eyeball (Tan et al., 2002). A recent development in the field has been the addition of deep learning (Pathirana et al., 2022). This has allowed the methods to improve on previous shortcomings such as low accuracy and generalizability to unconstrained environments and across people (Cheng et al., 2021).

Arguments in favor of computer-vision-based gaze estimation methods are typically based on their low initial cost, wide availability, and high scalability in terms of hardware (see, e.g., Krafka et al., 2016; Valliappan et al., 2020). First, cameras with video-recording capabilities can be quite cheap, especially when high spatial and temporal resolution are not required. Second, video cameras are already found nearly everywhere in modern society. They are used not only for consumer products such as smartphones and laptops, but also for surveillance, and are commonplace in many research settings as well. Third, cameras have a wide range of optics available. With a long-focus lens, a camera can be used to zoom in on a scene of interest, even from far away. This alone affords considerable freedom for where a camera can be placed to get a good view of the face. With these advantages, one can conceive many experimental situations where computer-vision-based gaze estimation could be applied, ranging from screen-based experiments to, for example, observations of parent–infant interactions. If the methods for computer-vision-based gaze estimation deliver what they promise, namely cheap, easily attainable, and accurate methods to estimate gaze, they could potentially be applied to the many situations where gaze behavior is of interest, or as Krafka et al. (2016) put it, “to bring the power of eye tracking to everyone” (p. 2183).

When examined from the perspective of someone without background or experience in computer science, however, the researcher interested in applying computer-vision-based gaze estimation is faced with two major problems. First, the developers of these methods either do not publish their code or publish it only for research-oriented purposes (namely, intended for other researchers in computer science) (Zhang et al., 2019). Second, the evaluations done thus far have largely been concerned with only absolute errors and comparisons with previous methods utilizing similar techniques (e.g., Baltrušaitis et al., 2018; Bao et al., 2021; Chen & Shi, 2018; Cheng et al., 2021; Chong et al., 2018; Fang et al., 2021; Krafka et al., 2016; Park et al., 2018; Wood & Bulling, 2014; Zhang et al., 2015, 2017), although a few notable exceptions with more comprehensive evaluations do exist (see Eschman et al., 2022; Kellnhofer et al., 2019; Valliappan et al., 2020; Zhang et al., 2019). In comparison, eye trackers bought from manufacturers are generally well documented. In addition, for many p-CR eye trackers, comprehensive performance evaluations even with regard to several specific populations are already readily available for researchers (e.g., Dalrymple et al., 2018; De Kloe et al., 2021; Hessels & Hooge, 2019; Morgante et al., 2012; Niehorster et al., 2020; and Table 2 from Holmqvist et al., 2022). When contrasted with what is available for p-CR eye tracking, the evaluations for computer-vision-based gaze estimation are severely lacking.

The goal of this paper is thus threefold. First, we introduce how computer-vision-based gaze estimation can be applied from the perspective of an experimental psychologist. Here, we define an experimental psychologist as someone with extensive experience conducting psychological experiments, but with very little or no experience in computer science. As it currently stands, it is not clear which methods are easily accessible and how they can be applied. Second, we provide a validation of the methods that can be used by researchers without expertise or a background in computer science. Third, we provide useful information for the developers of such methods so that they can become more aware of how their techniques can be improved and validated specifically for use in psychological research.

We evaluated computer-vision-based gaze estimation methods in two separate experiments. We then narrowed down the list of all the computer-vision-based gaze estimation methods we cite in this paper to those that (1) do not require calibration, (2) can be downloaded and installed without any required background knowledge in applying such methods, and (3) have proper documentation. This led to a final sample of two methods: those provided in the OpenFace (Baltrušaitis et al., 2018) and OpenGaze toolkits (Zhang et al., 2019). In the first experiment, we assessed the gaze estimation performance of the OpenFace and OpenGaze toolkits under optimal conditions, meaning experienced adult participants, good camera optics, and a task in which the head of the participant remains as stationary as possible without having to fix it in place with, e.g., a chin or forehead rest. We consider the methods viable under optimal conditions if they can be used to estimate the gaze position of the participant with good data quality. In the second experiment, we evaluated the gaze estimation performance of OpenFace in an experiment with infant participants. We were not able to assess the performance of OpenGaze in this experiment for several reasons, which we outline in the discussion. We consider OpenFace viable for infant gaze research if we can derive meaningful gaze-based measures that match closely to measures based on manual coding of gaze direction. The results and considerations of these two experiments will allow researchers interested in using computer-vision-based gaze estimation in fields such as psychology and educational science to better judge whether the tested methods might be suitable for their specific experiments.

## Experiment #1: Optimal conditions

### Method

#### Participants

A total of nine colleagues from the department of Experimental Psychology at Utrecht University participated in the experiment. The study was approved by the Ethics Committee of the Faculty of Social and Behavioural Sciences, Utrecht University (protocol number 21-0367).

#### Setup and stimuli

**Fig. 1:**
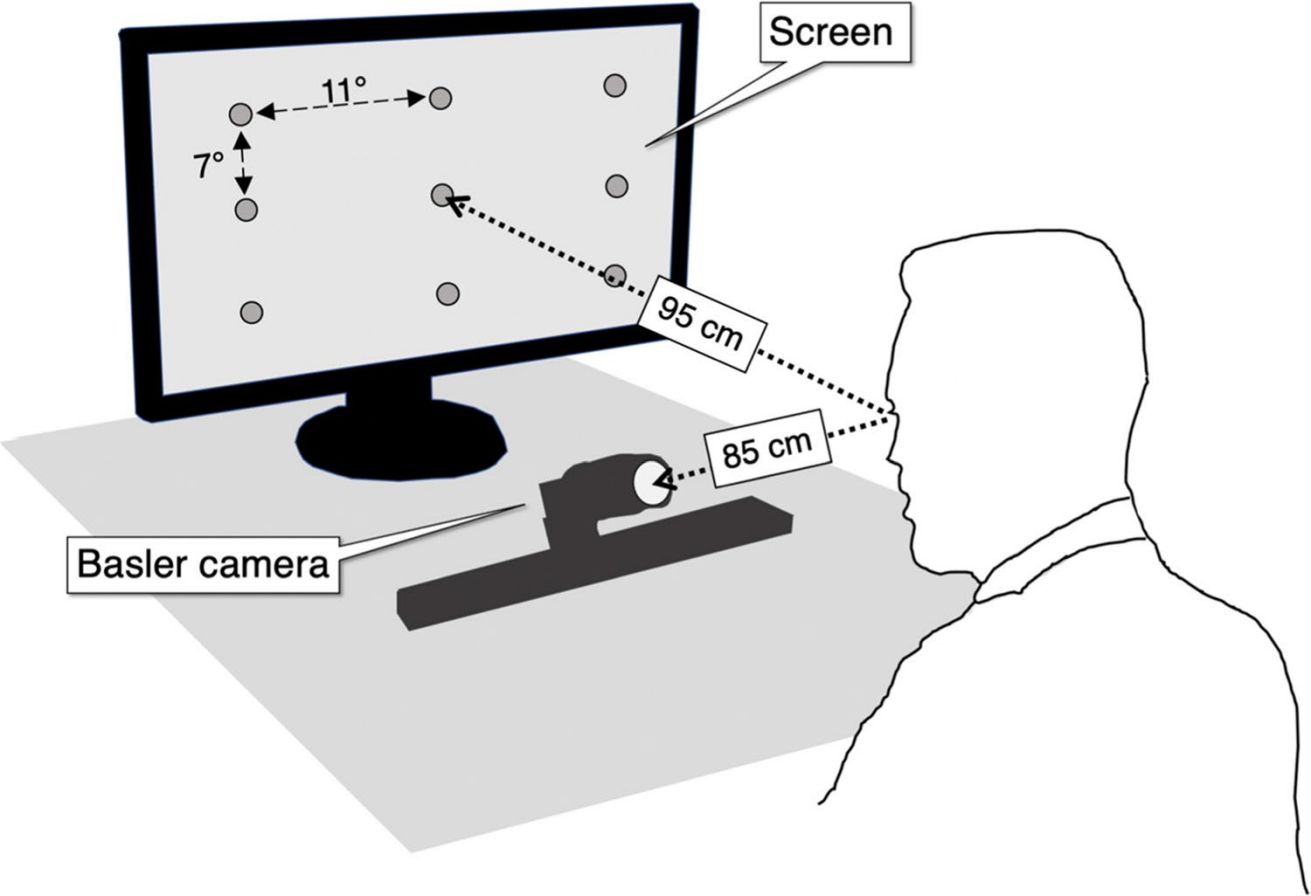
An illustration of the setup and stimuli for the first experiment. The Basler camera and computer screen to display the stimuli were placed in front of the participant at the distances specified in the figure. The participants were shown a grid of nine stimulus points one at a time in a left-to-right, top-to-bottom fashion. Assuming these fixed distances, the stimulus points on the grid were separated by a visual angle of roughly 11 degrees between adjacent points on the horizontal axis and 7 degrees between adjacent points on the vertical axis

A Basler ace acA2500-60um industrial camera equipped with a 25 mm Tamron C-Mount lens recording at a resolution of 2592 by 2048 pixels and a frequency of 60 Hz was used to record the participant’s face. It was placed on a table in front of the participant, filming from below with approximately 85 cm from the lens to the eyes. A 60 × 34 cm ASUS ROG computer screen using a resolution of 2560 by 1440 pixels was placed behind the camera at approximately 95 cm from the center of the screen to the eyes of the participant. There were some minor variations in these distances due to differences in participant heights. MATLAB was used to communicate with the camera (Hooge et al., 2021), and the Psychophysics Toolbox extensions (Brainard, 1997; Kleiner et al., 2007; Pelli, 1997) were used to present stimuli on the screen. The stimuli consisted of nine points with a distance of 18.6 cm between adjacent points on the horizontal axis and 11.6 cm between adjacent points on the vertical axis. When assuming the fixed positions we have described, these distances correspond to a difference in visual angle of roughly 11 and 7 degrees between adjacent points on the horizontal and vertical axis of the stimulus grid, respectively. The configuration and specific measures of the setup are illustrated in Fig. 1.

#### Procedure

Participants were first instructed to seat themselves in a chair placed in front of the computer screen and camera. The experimenter then adjusted the camera angle to make sure that the whole face of the participant was visible in the camera image. Next, the experimenter started the recording script in MATLAB. The first stimulus point was then presented in the top left corner. Participants were instructed to look at a set of points with only their eyes, starting from the first point already presented on the screen, while keeping their head fixated at the center, and to press the space key on the keyboard. This is hereafter referred to as the eyes-only condition. When the space key was pressed, the computer played a beep to signal that recording had started, then recorded a video for three seconds, followed by another beep to signal that recording had finished, after which the second point (i.e., the center top point) was presented on the screen. Each participant first fixated on all nine stimulus points in this fashion. After this, the experimenter instructed the participants to repeat the same procedure once. Next, the experimenter instructed the participant to again fixate on the points, but this time with both the eyes and the head, hereafter referred to as the head-and-eyes condition. The procedure was similarly completed twice for all nine points. As each condition was done twice per participant, the experiment resulted in two recordings per participant per condition. With nine participants, this totaled 18 recordings per condition.

#### Gaze data

As mentioned in the introduction, the only computer-vision-based gaze estimation methods we found to fulfill our selection criteria were those provided in the OpenFace (Baltrušaitis et al., 2018) and OpenGaze (Zhang et al., 2019) toolkits. We further considered Gaze360 (Kellnhofer et al., 2019), for which the developers also provide step-by-step instructions for installation and use of a (beta version of a) demo code. Unfortunately, we were not able to successfully install the software even when following the step-by-step instructions and testing on multiple systems. Gaze360 was therefore not included in our analyses. OpenFace reports eye gaze direction estimates in world coordinates averaged for both eyes. In the OpenFace wiki, it is stated that looking straight forward should result in a gaze angle of roughly zero degrees on both axes, while looking from left to right should result in a change in gaze angle from positive to negative on the horizontal axis, and looking from top to bottom should result in a change in gaze angle from negative to positive on the vertical axis. OpenGaze, on the other hand, reports estimated gaze position in normalized coordinates. For further explanation of the different coordinate systems used for gaze estimation, we refer the reader to Pathirana et al. (2022).

### Results

Our goal was to assess the gaze estimation performance of the computer-vision-based gaze estimation methods that filled our criteria. We examined the performance of the methods by computing three commonly used measures to assess eye-tracking data quality: accuracy, precision, and data loss (Holmqvist et al., 2022). To note, we ran all analyses separately with data from only the first recording from all participants, with data from only the second recording from all participants, and with data from both recordings from all participants. Varying the number of recordings used per participant did not change our conclusions. Therefore, we used all 18 recordings per condition for the reported analyses.

#### Were the gaze estimates sufficiently accurate?

In eye-tracking research, accuracy is used to represent how close the estimated gaze position is to the true gaze position. To determine the accuracy of the gaze estimates in the present study, we would need to know the true (physical) position of each stimulus point in the world as well as the estimated gaze position for when each participant fixated on each point in the same coordinate system. The coordinate system of neither method was sufficiently clear for us to be able to do this. In the OpenFace output format wiki, it is stated that the estimated gaze angles represent eye gaze direction in world coordinates, converted into a format that is easier to use than gaze vectors. Despite a comprehensive output format wiki, examining absolute accuracy was not possible, as we were not able to determine what the origin of the coordinate system was and could therefore not compare the estimated gaze angles directly to the physical gaze angles observed in the setup. For OpenGaze, we were not able to find out what the assumed geometry of the setup was or how a custom setup could be defined. Because of this, it was not possible to directly relate the normalized coordinates to our stimulus grid. Thus, to answer our question, rather than computing absolute accuracy, we examined it on the level of the configuration and scale of the nine fixations as represented in the gaze estimates of both methods.

**Fig. 2:**
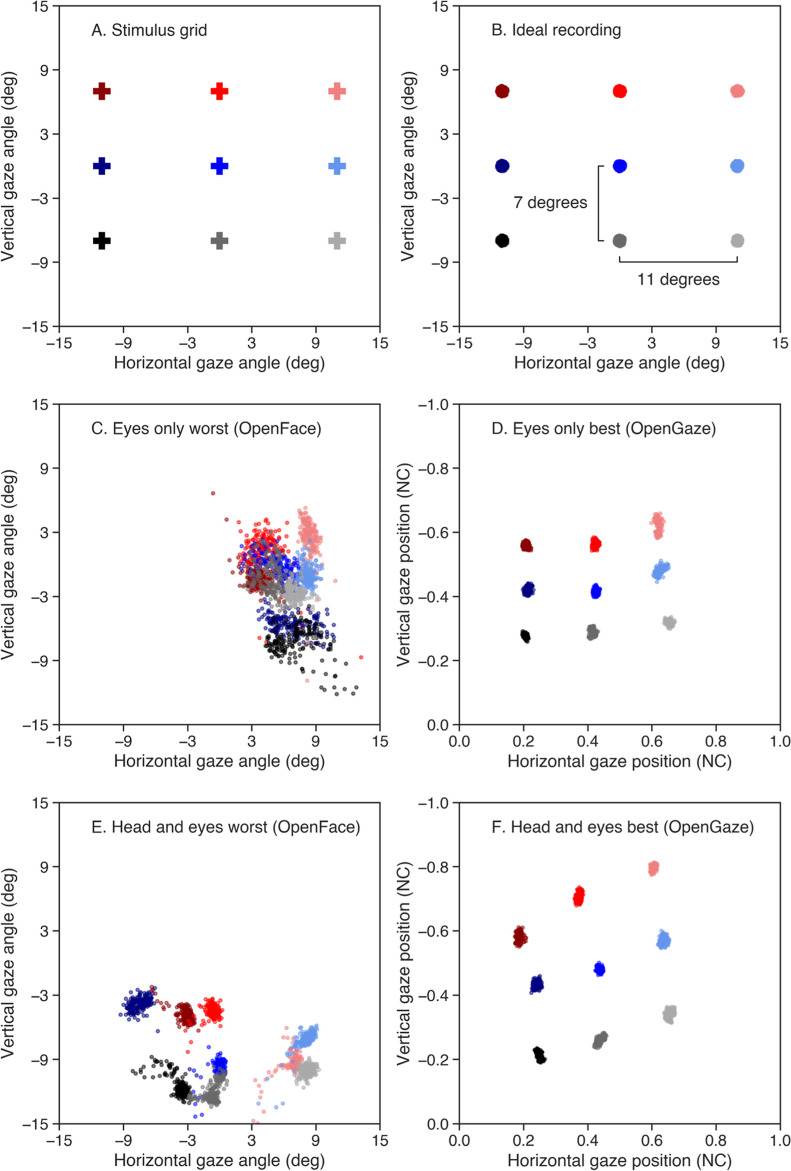
The expected, best, and worst recordings. The different colors represent the three rows of the grid, while the shades of the colors represent the three columns. Estimates for OpenFace were in degrees of gaze angle, while estimates for OpenGaze were in normalized coordinates (NC). **A** The stimulus grid presented to the participants. **B** The ideal recording with little variation within fixations and a similar scale to the stimulus grid. **C** The worst recording for the eyes-only condition across both methods (an OpenFace estimate). **D** The best recording for the eyes-only condition across both methods (an OpenGaze estimate). **E** The worst recording for the head-and-eyes condition across methods (an OpenFace estimate). **F** The best recording for the head-and-eyes condition across methods (an OpenGaze estimate). Note that the sign of the gaze estimates for OpenFace has been flipped for increased interpretability. Looks from left to right and down to up result in a change in gaze angle from negative to positive

For the methods to be accurate on the level of the configuration, we would expect to be able to reliably distinguish between the nine fixations when plotting the horizontal and vertical gaze estimates of a recording against each other. Figure 2A represents our stimulus grid with the nine stimulus points on which participants fixated. Figure 2B represents what we would expect to see in the gaze estimates of an ideal recording, meaning nine clusters representing the nine fixations in a similar configuration as in our stimulus grid. Figure 2C–F illustrate the best and worst recordings as visually judged by the researchers. The best and worst recordings were defined as most and least visually similar to the ideal recording (Fig. 2B) in terms of separation between fixations, spread within fixations, and the orientation of the complete configuration of fixations. As Fig. 2C–F illustrate, it was possible to distinguish between the nine fixations for some but not all recordings. In the worst case, it was not possible to distinguish between any fixations (Fig. 2C). In the best case, however, the nine fixations could clearly be seen in the plotted gaze estimates. Notably, the best observed configuration for both conditions was with OpenGaze estimates (Fig. 2D and F), while the worst observed configuration for both conditions was with OpenFace estimates (Fig. 2C and E).

**Fig. 3:**
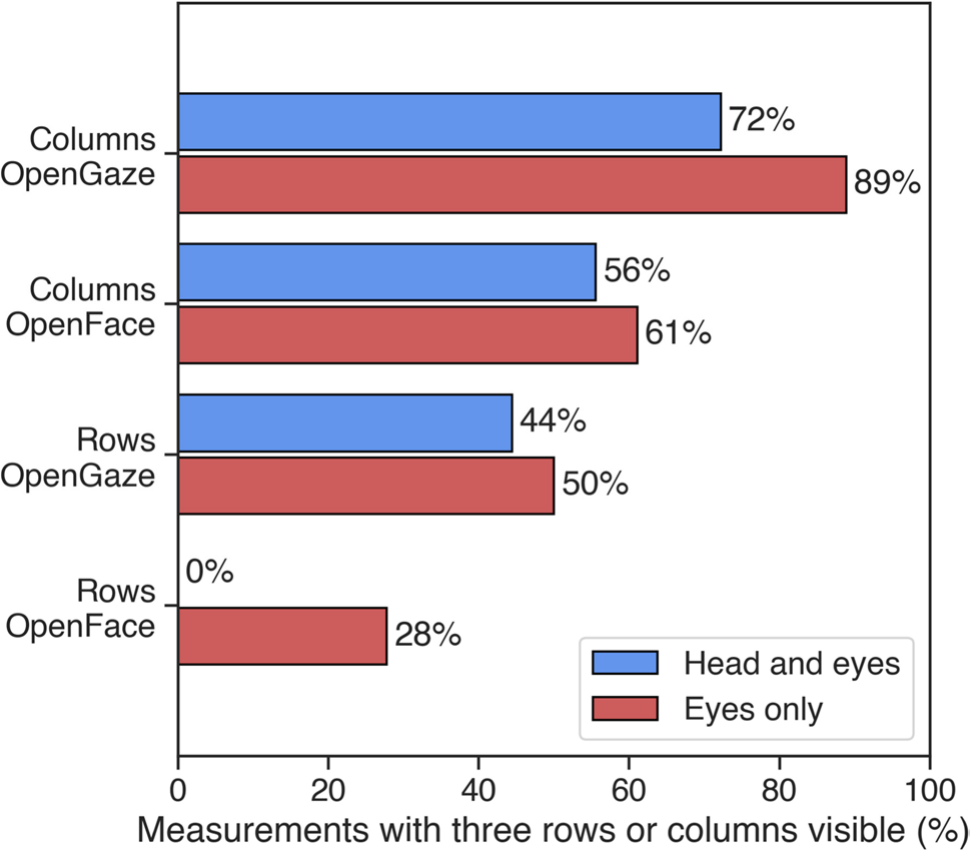
The three rows and columns of fixations as observed in the gaze estimates. The blue bars stand for the head-and-eyes condition while the red bars stand for the eyes-only condition. Values on the horizontal axis represent the percentage of recordings per condition in which the three rows or three columns of fixations could clearly be distinguished in the gaze estimates by the two methods

For OpenFace, three columns of fixations could be seen in the gaze estimates for 61% (11) of the 18 recordings for the eyes-only condition and in 56% (10) of the recordings for the head-and-eyes condition. Three rows of fixations could only be seen in 28% (5) of the 18 recordings for the eyes-only condition and for 0% (0) of the recordings in the head-and-eyes condition. OpenGaze performed better, as three columns could be seen in 89% (16) and 72% (13) of the 18 recordings for the eyes-only and head-and-eyes conditions, respectively. Three rows could be seen in 50% (9) of 18 recordings for the eyes-only condition and in 44% (8) for the head-and-eyes condition. For both methods, when it was possible to distinguish between three columns of fixations, it was also possible to distinguish between three rows of fixations, meaning that the complete grid of stimulus points was clearly represented. These results are illustrated in Fig. 3.

**Fig. 4:**
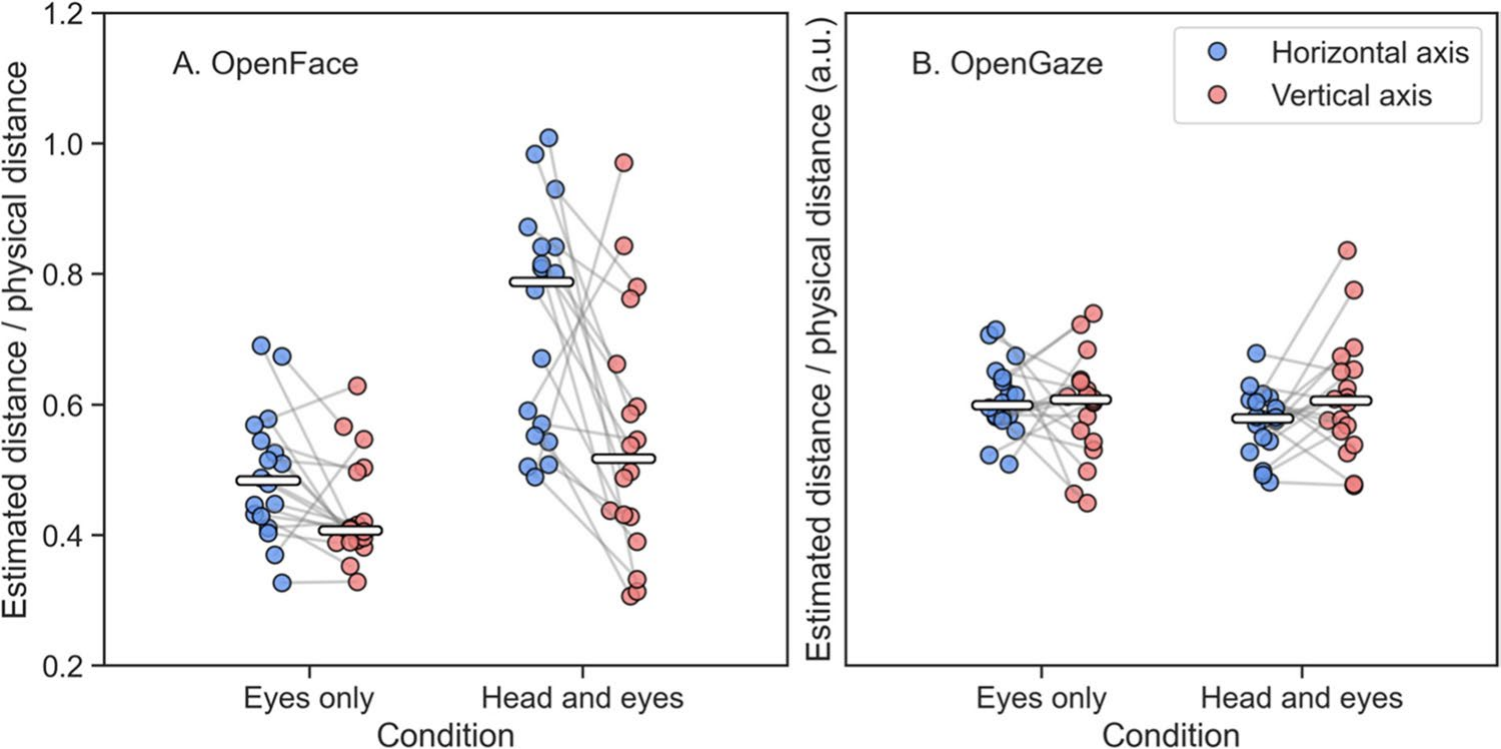
Distance between adjacent fixations divided by the physical distance between stimulus points on the stimulus grid for **A** OpenFace and **B** OpenGaze estimates. For panel A, the vertical axis of the plot indicates the ratio of the median angular difference between adjacent fixations to the angular difference between adjacent points on the stimulus grid. The blue circles represent values for the horizontal axis, while the red circles indicate the same for the vertical axis. The horizontal white lines represent the median ratios. The gray connecting lines indicate that the values belong to the same recording. A ratio of 1 indicates that the fixations accurately represented the stimulus grid in terms of scale, while a ratio less than or greater than 1 indicates that the distance between adjacent fixations differed from the distance between points on the stimulus grid. Panel B represents the same for estimates provided by OpenGaze. As the OpenGaze estimates could not be related to the stimulus grid for reasons discussed in the text, values on the vertical axis of the plot are not meaningful and have been omitted. Panel B should only be examined in terms of the differences between the two axes and two conditions

Next, we examined the accuracy of the methods in terms of scale. For the scale of the estimates to be accurate, we would expect the distances between fixations to resemble the distances between points on the stimulus grid. Examining the scale was possible for OpenFace estimates, as we could directly compare angular separation between fixations to angular separation between stimulus points. Points on the stimulus grid were spaced 11 degrees horizontally and 7 degrees vertically with respect to the participant’s eyes. Therefore, in an ideal recording (Fig. 2B), we would expect to see an 11-degree difference in gaze angle between adjacent horizontal fixations and a 7-degree difference in gaze angle between adjacent vertical fixations. To determine whether this was the case, we first computed the median horizontal and vertical OpenFace gaze angle for each fixation. We then further calculated the median angular distance between all adjacent fixations for each axis and divided it by the corresponding angular distance between the points on the stimulus grid (either 11 or 7 degrees, depending on the axis). The ratio obtained represents the accuracy of the estimates provided by OpenFace in terms of scale. A ratio of exactly 1 indicates an accurate representation of the stimulus grid, while ratios greater than 1 indicate overestimation and ratios less than 1 indicate underestimation. We found that when fixating with only the eyes, the distance between adjacent fixations was less than the distance between points on the stimulus grid (see Fig. 4A). The ratios for both axes ranged from roughly 0.3 to 0.7, and the median ratio was lower for the vertical axis. When fixating with the head and eyes, the results differed by a larger margin, with ratios ranging from roughly 0.5 to 1 for the horizontal axis and from roughly 0.3 to 1 on the vertical axis, as well as a greater difference in median ratio between the axes. Similar comparisons were not possible for OpenGaze estimates, as they were in normalized coordinates and could not be directly related to the angular separation of the stimulus grid. We elaborate on this in more detail in the general discussion. Nevertheless, it was still possible to examine whether there was a difference in the accuracy of the estimates in terms of scale between the two axes and two conditions. To this end, we performed the same calculations with the OpenGaze estimates. These results are shown in Fig. 4B. Based on the plotted data, it appears that estimates by OpenGaze were more consistent across axes and across conditions than their OpenFace counterparts; OpenGaze seems to report gaze estimates in a similar scale regardless of whether one fixates with only the eyes or with both the head and the eyes for both the horizontal and vertical axis.

Taken together, the estimates for both OpenFace and OpenGaze were more accurate in the horizontal direction than in the vertical direction. It was possible to distinguish between three columns of fixations for over half of the recordings for OpenFace and for nearly all recordings for OpenGaze. For the vertical direction, there was more overlap between fixations for both OpenFace and OpenGaze, hence making it more difficult to distinguish three rows of fixations in the plotted data. This can be at least partly explained by the smaller difference in gaze angle between the stimulus points in the vertical direction. Importantly, there was high variability in the accuracy of the estimates in terms of scale between the conditions for OpenFace but not for OpenGaze, indicating that for OpenFace, the gaze estimates were affected by head rotation, while OpenGaze produced similar estimates regardless of whether one fixated with the eyes only or with both the head and the eyes. OpenFace underestimated gaze angles for both conditions, while for OpenGaze it was not possible to determine whether there was underestimation or overestimation. On average, the estimates were accurate enough to distinguish between fixations to the nine stimulus points in the horizontal direction for OpenGaze but not for OpenFace. As variability between the two axes and conditions for OpenGaze was low, we conclude it to also be sufficiently accurate on the vertical axis, as long as there is at least a difference of 11 degrees of gaze angle between stimulus locations, as was the case for the horizontal axis. Estimates by OpenFace were much more variable and not accurate enough in the vertical direction.

#### Were the gaze estimates sufficiently precise?

Precision refers to how consistent the reported gaze estimates are. If the estimates by the methods are precise, we would expect that when a participant fixates on a point presented on a screen, the reported gaze estimates over the duration of the fixation should be close to each other. In eye-tracking research, precision is typically operationalized by either the standard deviation or the sample-to-sample root mean square of the gaze signal (Holmqvist et al., 2012). A reasonably precise recording would look like the ideal recording presented in Fig. 2B, where there is little variation in the gaze estimates within fixations. As Fig. 2D and F illustrate, precision was high for the best recordings for both conditions, as variation within fixations was low. In the worst recordings (Fig. 2C and E), there was considerable variation within fixations. As the worst recordings illustrate, low accuracy combined with low precision can lead to major overlap between fixations.

**Fig. 5:**
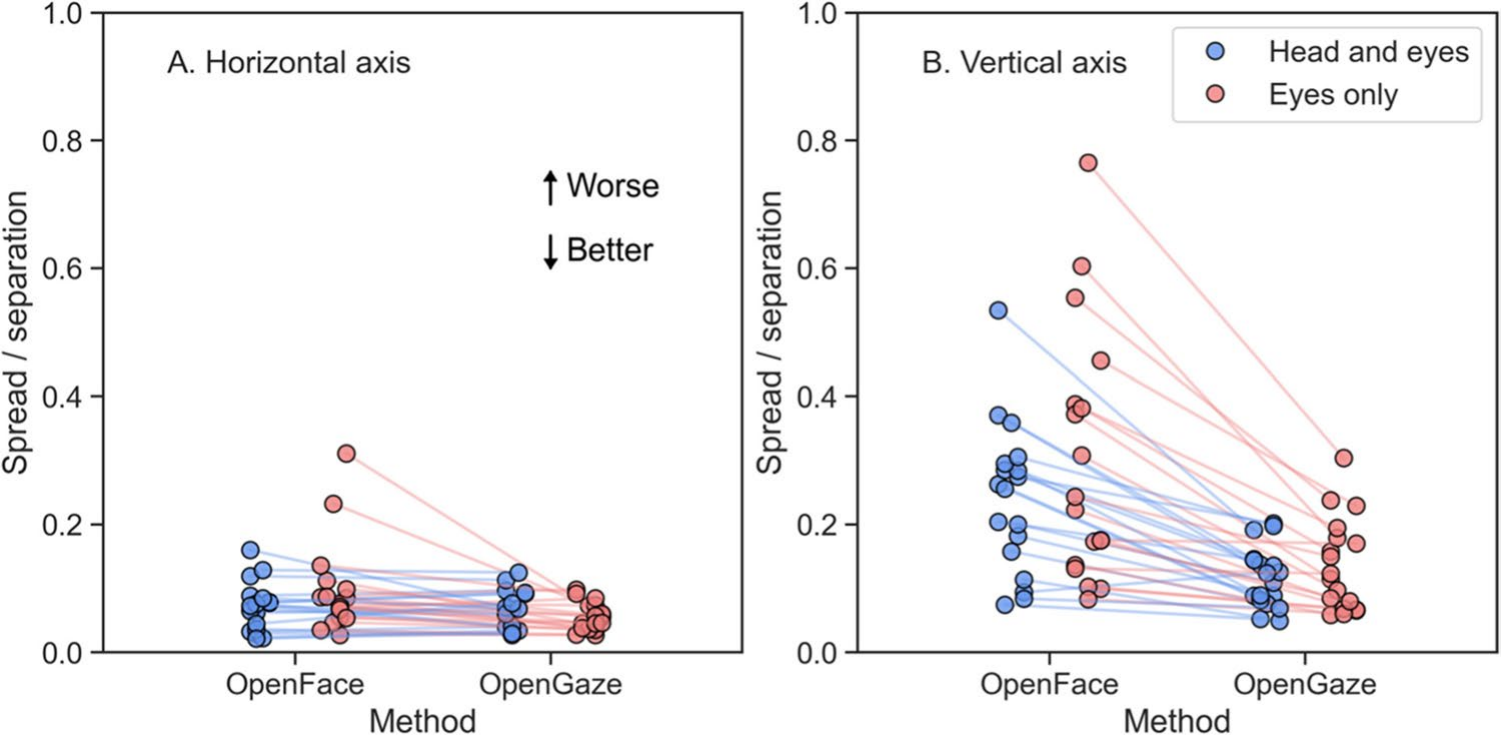
Spread within fixations divided by separation between fixations for **A** the horizontal axis and **B** vertical axis. The blue and red circles stand for individual recordings in the head-and-eyes and eyes-only conditions, respectively. The connecting lines indicate that the ratio was computed using estimates derived from the same recording. The horizontal axis of the plot indicates the method used to process the recording, while the vertical axis represents the ratio of spread to separation for the fixations. Values close to zero indicate that it is possible to distinguish that the observer is fixating on a specific point on the grid, while higher values indicate the opposite

To examine precision relative to the estimated grid of fixations, we divided the median spread (i.e., the standard deviation of the gaze signal) within fixations on each axis by the median distance between fixations on that axis for each recording. A resulting ratio close to zero indicates that distinguishing between fixations on that axis was generally possible. Conversely, a higher ratio means that it is difficult to distinguish between fixations on that axis and overlap is likely. For the horizontal axis (Fig. 5A), the ratios for both methods and both conditions were similarly low (except for two OpenFace recordings, both belonging to the same participant), suggesting that there was little overlap between adjacent fixations. Ratios for the vertical axis were higher (Fig. 5B), indicating more overlap between adjacent fixations. This was expected because the stimulus points on the vertical axis were closer to each other than the stimulus points on the horizontal axis. There was substantially more variability in the ratios between the methods for the vertical axis; ratios for OpenFace were much higher than those for OpenGaze.

Based on visual examination of the spread within fixations as well as the low variation in the ratios of spread to separation between the two conditions, we conclude that average precision in the horizontal direction was sufficient for both methods. For OpenGaze, precision in the vertical direction was similar to precision in the horizontal direction, and variability between conditions was low for precision on both axes. For OpenFace, precision in the vertical direction was substantially lower than precision in the horizontal direction, and variability in the ratios of spread to separation between conditions was high. Thus, for estimates in the vertical direction, precision for OpenGaze was sufficient but the precision for OpenFace was not.

#### Were there any lost data?

**Fig. 6:**
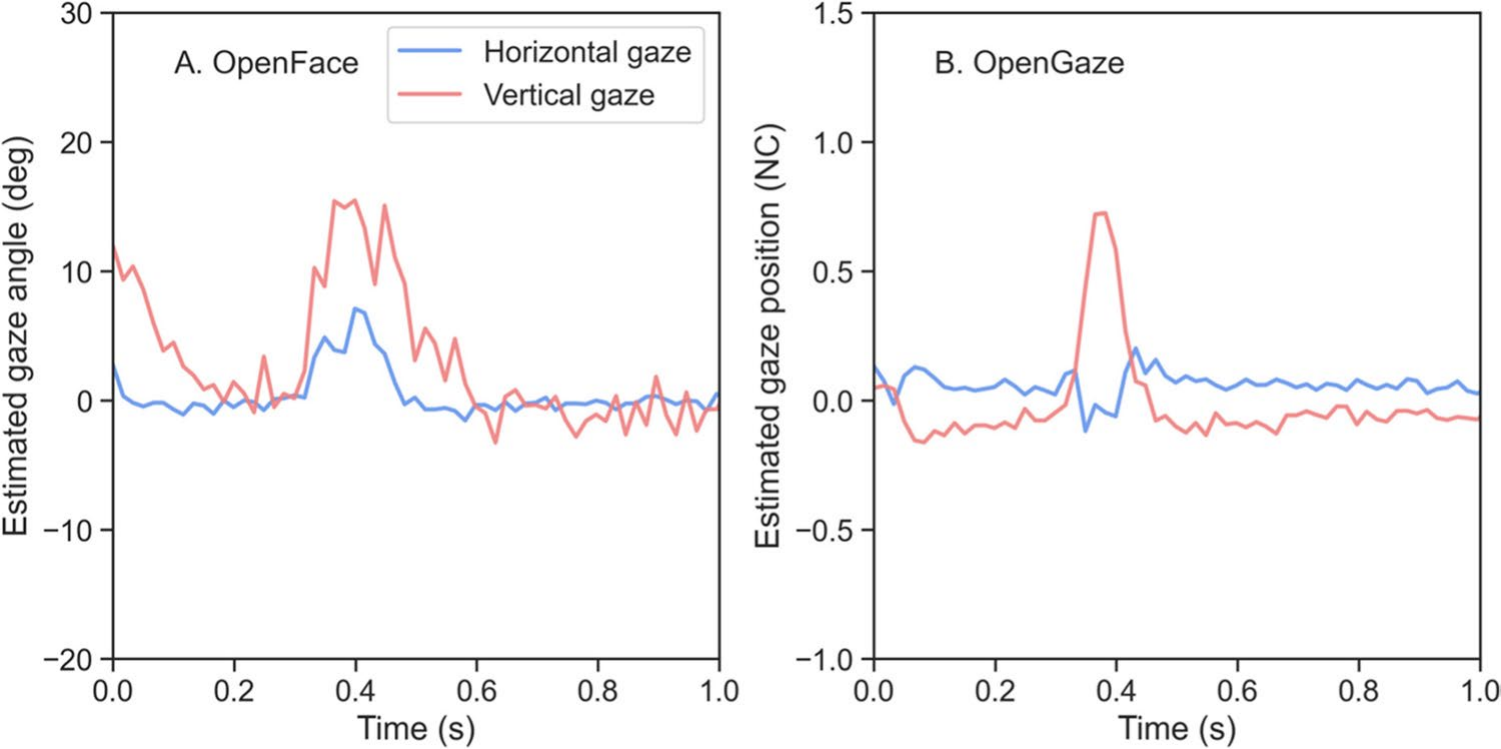
An example of a blink represented in **A** OpenFace and **B** OpenGaze estimates. The blue and red lines represent the horizontal and vertical gaze signals, respectively. The horizontal and vertical signals were repositioned to be centered around roughly zero for plotting purposes. The horizontal axis of the plot denotes time in seconds, and the vertical axis denotes estimated gaze angle in either degrees or normalized coordinates (NC). Note that the sign of the gaze estimates for OpenFace has been flipped for increased interpretability. Looks from left to right and down to up result in a change in gaze angle from negative to positive

Another important aspect of data quality in eye-tracking research is data loss. Data loss refers to how much missing data there were for a given recording. Data loss could be due to error in the recording tool (e.g., when the participant blinks or when an eye tracker is not able to find the eyes even when they are within the area the eye tracker is capable of measuring), or due to the behavior of the participant (e.g., when the eyes or the face of the participant has moved outside the area the eye tracker is able to measure). The participants in our experiment were always facing the camera and a video of their face was only being recorded when they were fixating on the points, meaning that the eyes and face were always visible to the camera. Any data loss should be due solely to recording error, or if, for some reason, the eyes of the participant were not visible to the camera (e.g., when the participant blinks). We computed the percentage of data loss, defined as the number of samples with a missing gaze estimate, for all 36 recordings (18 per condition). There were no lost data in any of the recordings for either method. Upon examining the recorded videos, however, we found that a few participants blinked on a few occasions when fixating on the points. Notably, both OpenFace and OpenGaze reported gaze estimates even for periods when the eyes of the participant were fully closed in the video recording. When the eyes were closed, both methods showed a sharp change in the gaze estimate on the vertical axis (see Fig. 6). It thus appears that with a camera filming the face from below under optimal conditions, data loss does not seem to be a problem for either method. Blinks can potentially be identified by sharp changes in the velocity of the vertical component of the gaze signal.

### Interim summary

Participants fixated on nine stimulus points presented on a computer screen while a camera recorded a video of their face. The videos were processed using the OpenFace and OpenGaze toolkits. We investigated the accuracy, precision, and percentage of data loss of the gaze estimates. In summary, the accuracy and precision of the estimates by OpenGaze were sufficient to distinguish between fixations on the horizontal axis for nearly all recordings. The estimates by OpenFace, on the other hand, were only accurate enough to distinguish between the points for a bit over half of the recordings. Importantly, OpenGaze estimates were resilient to changes in looking behavior (i.e., fixating with only the eyes vs. fixating with both the head and eyes) while OpenFace was not. Data loss was sufficiently low for both methods. We conclude that OpenFace and OpenGaze can potentially be used in sparse environments (e.g., three relatively large areas of interest [AOIs]) with horizontally separated stimuli. OpenGaze can possibly also be used in less sparse environments and for both horizontally and vertically separated stimuli, with stimuli separated by at least 11 degrees of gaze angle with respect to the participant’s eyes.

## Experiment #2: Infant participants

In the second experiment, we examined whether computer-vision-based gaze estimation can be used to derive meaningful dwell-based measures from the data concerning infant participants. For this experiment, only OpenFace gaze estimates were evaluated. We address the reasons for this in the general discussion.

### Method

#### Participants

The sample consisted of 44 approximately 10-month-old infants, who were part of a larger longitudinal study on infant siblings of children with autism (Early Autism Sweden, EASE, http://www.smasyskon.se). Participants were recruited mainly from the greater Stockholm area, either through the project’s website, advertisement, and clinical units (infants with an older sibling with autism) or through birth records and advertisements (infants with a typically developing older sibling). Participants are typically seen at 5, 10, 14, 18, 24, and 36 months, but the data being analyzed here were from the 10-month time point only. The infants included in the study were reported by their parents to have no known medical conditions such as epilepsy, no known genetic syndrome associated with autism nor any other known medical conditions affecting brain development, and no visual or auditory impairment, and were born full-term (after week 36). Although the majority were at elevated likelihood for autism, meaning an expected larger heterogeneity of later developmental trajectories, we reasoned that the sample would work well for the present methodological purposes. Although some of these children are likely to later develop symptoms of autism or other neurodevelopmental conditions, the group is not very atypical in terms of basic eye movements, at least not in any sense that would prevent the current investigation.

#### Setup and stimuli

The setup consisted of two Logitech Brio web cameras connected to one operating computer running Windows 10. Infants sat in a baby chair across the table from their parent. The cameras were placed on the corners of the table on the side of the parent, approximately 120 cm from the infant’s face, angled in such a way that the face of the infant was centered in both camera images. The cameras were operated using a Python script utilizing OpenCV. For each camera, a text file denoting the system timestamp for each frame of the recording was saved. Each camera recorded at approximately 30 Hz. A lamp that emitted light and rotated when turned on was placed on each side of the parent. Importantly, it was a sparse environment with attractive stimuli from the perspective of the infant, making it ideal to hand-code the gaze direction.

**Fig. 7:**
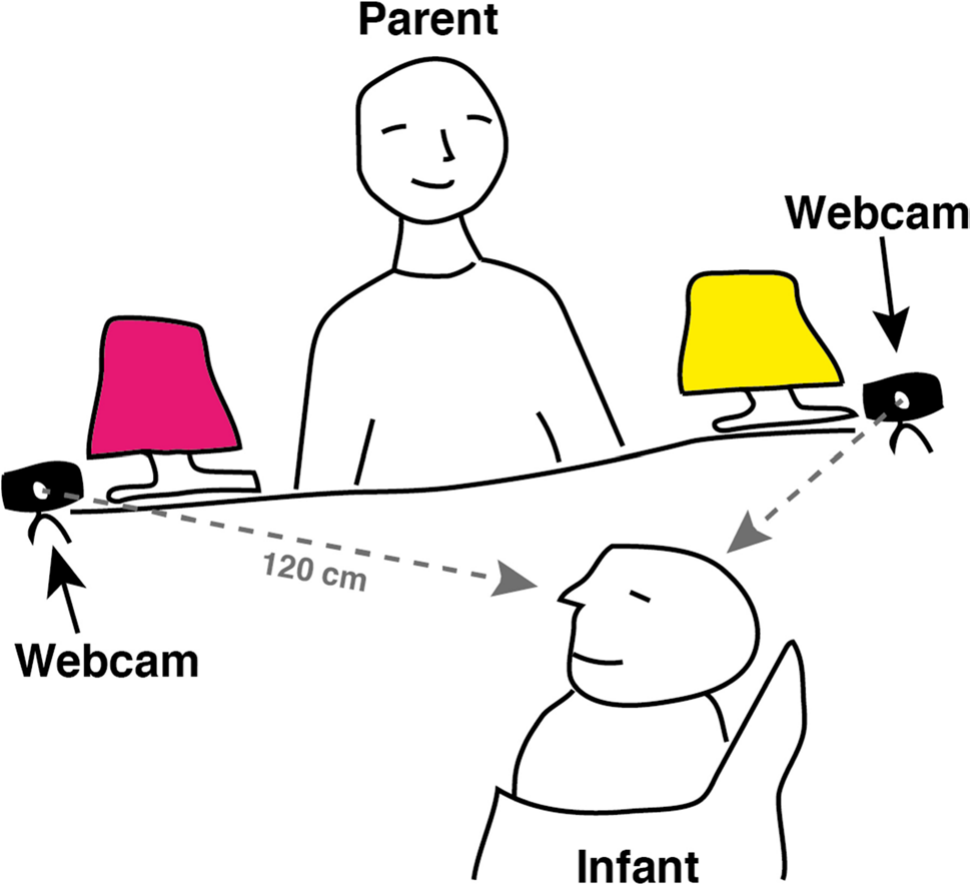
Illustration of the setup for the second experiment. The infant was seated in a baby chair on one side of a table, with the parent on the other side. One lamp was placed on each side of the parent. Two Logitech Brio web cameras placed on the outer side of each lamp were used to record a video of the face of the infant at approximately 120 cm from the infant’s face

Pilots conducted prior to the experiment revealed that the best OpenFace estimates could be obtained when placing the camera directly in front of the participant, filming their face from below, as is typically done with eye trackers. However, this was not possible, as this experiment was part of a longer series of experiments which involved the parent manipulating objects directly in front of them. Placing the camera in front of and below the face of the infant would have been in the way for those experiments. After examining the quality of the estimates using different camera positions, we opted for a setup with two web cameras filming the infant from both sides of the parent, with the intention of averaging the gaze estimates from both cameras, as this produced the best estimates given the limitations regarding camera placement. The setup is illustrated in Fig. 7.

#### Procedure

Upon arriving in the experiment room, the infant was first seated on one side of the table and the parent on the other. The parent was provided with a box with instructions on what to do in the experiment. For this part, the parent read a note saying “Something will now happen on the table that you and the child can look at.” After that, one of the lamps lit up and started rotating for five seconds. The lamp was then shut off, and nothing happened for ten seconds. Thereafter, the other lamp lit up and rotated for five seconds, after which nothing happened for ten seconds. This pattern was repeated for 90 seconds.

#### Gaze data

Infant gaze data were sourced using two methods: manual coding of gaze direction and OpenFace. Manual coding was done by author NVV based on the recorded videos from both cameras using a Python script employing OpenCV. The scene in front of the infant contained three major areas of interest (AOIs): the left lamp, the parent, and the right lamp. A dwell at an AOI was defined to start at the first frame where the eyes of the infant (based on pupil direction) were oriented towards the AOI. Dwells were defined to end at the first frame where the eyes of the infant were oriented toward a new AOI. If the gaze was not on any of the three AOIs, it was assigned to a “none” AOI. This resulted in a data file with the start and end time of each dwell for each participant.

To extract OpenFace gaze estimates, we initially processed the recordings from both cameras with the intention of averaging the gaze signal from them. Upon inspection of the data, we found that averaging would not be possible for many participants as often the recording from the camera on the right side of the parent was fuzzy or the cameras were angled differently. OpenFace gaze estimates based on the recordings from the camera on the left side of the parent were better on average. Therefore, we restricted all our analyses to estimates based on the recordings of that camera only.

#### Assigning OpenFace gaze estimates to AOIs

**Fig. 8:**
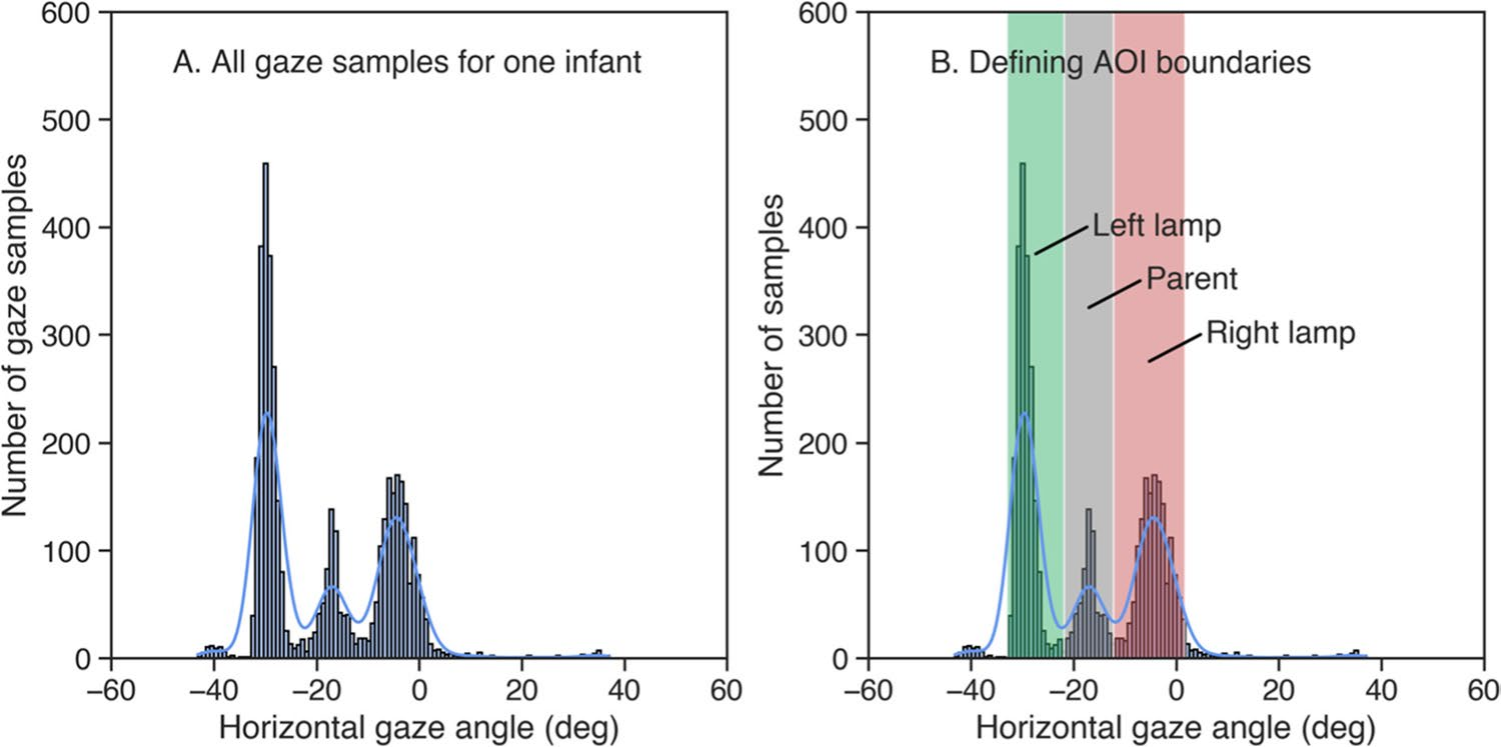
An example of **A** plotting a histogram based on the horizontal component of all gaze samples for one participant and **B** AOI assignment based on the peaks observed in the histogram. In both panels, values on the horizontal axis represent the horizontal gaze angle in degrees, while values on the vertical axis represent the number of samples within one bin. Panel A depicts a histogram derived from the horizontal component of all gaze samples from one participant with three peaks clearly visible. Panel B depicts the AOI assignment based on the boundaries of the peaks. Values falling within the green color were assigned to the “left lamp” AOI, values within the gray color were assigned to the “parent” AOI, and values within the red color were assigned to the “right lamp” AOI. Values not falling under any of these AOIs were assigned to a “none” AOI. Note that the sign of the gaze estimates has been flipped for increased interpretability. Looks from left to right result in a change in gaze angle from negative to positive

To calculate dwells to the different AOIs for the OpenFace estimates, we first had to define the boundaries of the AOIs in terms of OpenFace gaze angles. As the OpenFace coordinate system was not sufficiently clear to us, we adopted a similar technique to that used in Nyström et al. (2019), meaning defining AOI boundaries based on the spatial distribution of the estimates. To achieve this, we first plotted a histogram of all the gaze estimates over the whole experiment separately for each participant (see Fig. 8A). This was done using only the horizontal gaze angles, as the AOIs were horizontally separated and OpenFace had been shown to perform worse in the vertical direction in the first experiment. With three horizontally separated AOIs, we expected to observe three peaks in the gaze estimates of each infant: one for the left lamp, one for the parent, and one for the right lamp. The peak for the parent AOI was assumed to be located between the peaks for the left and right lamp, as was the case in the configuration of the setup (Fig. 7). With this assumption, we examined the histogram of the horizontal gaze estimates for each participant and divided it into three sections that most closely resembled three peaks, as illustrated in Fig. 8B. The left peak was assumed to represent the “left lamp AOI.” The center peak was assumed to represent the “parent” AOI. The right peak was assumed to represent the “right lamp” AOI. Given that the lamps were designed to be engaging stimuli to infants, we assumed that they would be looked at the most. Therefore, if only two peaks were present in the histogram, the space between the peaks was assumed to belong to the parent AOI. If there were less than two discernible peaks in the histogram, the data for the participant were excluded from all further analyses. As shown in Fig. 8B, the boundaries of the divided sections were then used to assign each sample of gaze data to its respective AOI. If a gaze sample was not within the boundaries of any section, it was assigned to a “none” AOI. Following, we calculated dwells to the AOIs for each participant. Dwells based on OpenFace estimates were defined to start at the first frame where the gaze was on an AOI and to end when the gaze was on another AOI for at least three consecutive frames. The purpose of this was to prevent dwells from being broken prematurely.

### Results

The purpose of the second experiment was to determine whether computer-vision-based gaze estimation could be used to derive meaningful gaze-based measures in a sparse environment with infant participants and horizontally separated stimuli. The original sample consisted of 44 infants. Fourteen infants were excluded because it was impossible to manually code the videos for gaze direction because of, e.g., missing data, camera error, or the experiment ending prematurely. Five further participants were excluded due to there being less than two discernible peaks in the histograms of the horizontal gaze estimates, leading to a final sample of 25 infants.

#### Were OpenFace-based measures similar to manual coding?

**Fig. 9:**
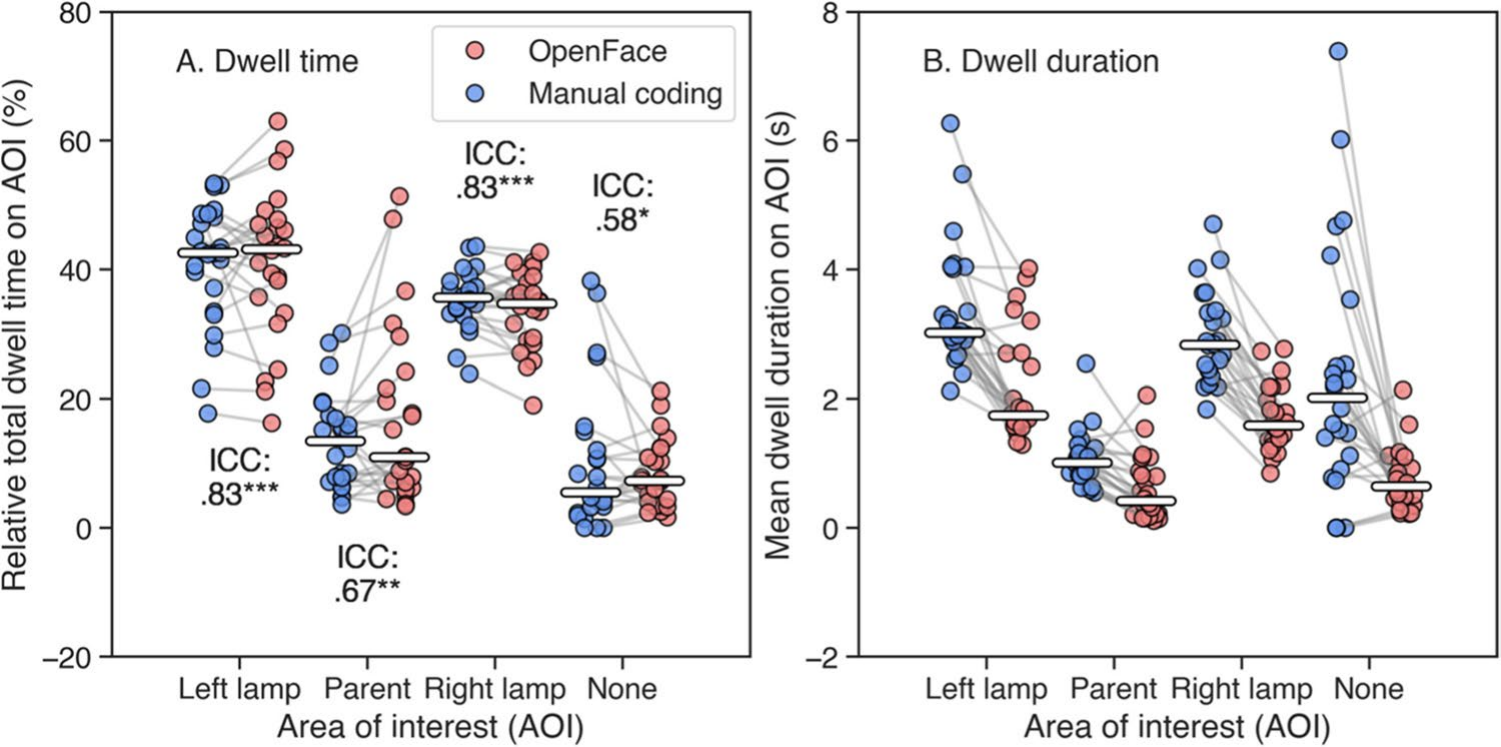
Comparison of OpenFace-based estimates and manual coding of gaze direction for **A** total dwell time and **B** dwell duration. The red circles represent values for individual participants based on OpenFace gaze estimates, while the blue circles represent values based on manual coding of gaze direction. The gray lines indicate that the measures were derived from the same recording. The horizontal white lines represent the median values. The horizontal axis for panel A represents the four different AOIs, and the vertical axis represents the relative total dwell time on them. ICC values are displayed when significant, with the number of asterisks representing level of significance (* *p* < .05, ** *p* < .01, *** *p* < .001). The horizontal axis for panel B represents the four different AOIs, while the vertical axis represents the mean dwell duration on them

To compare OpenFace estimates with manual coding, we first computed the relative total dwell time on each AOI for each participant. These results are plotted in Fig. 9A. As can be seen in the figure, median relative total dwell times on the “left lamp,” “parent,” “right lamp,” and “none” AOIs were quite similar for both OpenFace and manual coding. When looking at the individual level by examining the grey lines connecting the relative total dwell times derived using the same recording, the difference was quite large for some participants and very small for others. This was more pronounced for the “parent” and “none” AOIs. To examine the differences further, we calculated the intraclass correlation coefficients (ICC A1; see McGraw & Wong, 1996; Weir, 2005) for absolute agreement between OpenFace and manual coding for each AOI separately. These calculations revealed a significant correlation for all four major AOIs. The ICC value for the “left lamp” AOI was .83 with a 95% confidence interval (CI) of .60 to .92 (df1 = 23, df2 = 24, *p* < .001), indicating moderate to excellent reliability according to the guidelines by Koo and Li (2016). For the “parent” AOI, ICC was .67 with a 95% CI of .25 to .86 (df1 = 23, df2 = 24, *p* = .005), indicating poor to good reliability, and for the “right lamp” AOI ICC was .83 with a 95% CI of .61 to .92 (df1 = 23, df2 = 24, *p* < .001), indicating moderate to excellent reliability. ICC for the “none” AOI was .58 with a CI of .03 to .82 (df1 = 23, df2 = 24, *p* = .02), indicating poor to good reliability.

To further examine how OpenFace estimates compared with manual coding of gaze direction, we calculated the mean dwell duration for each AOI for each participant. Mean dwell duration gives an indication of how long participants looked at a specific AOI on average. As Fig. 9B shows, mean dwell duration based on manual coding was greater than mean dwell duration based on OpenFace estimates for all AOIs. Given that the relative total dwell times were similar, this means that OpenFace-based dwells were shorter and that there were more of them on average. We further calculated the ICC values for the dwell durations as well, but no significant correlations were found, indicating poor reliability on average. This can potentially be explained by the difference in how dwells were determined between the two methods. For manual coding, the coder could determine the start and end of a dwell directly based on when the gaze of the infant was observed to be on an AOI and when it shifted to a new AOI. With the context before and after dwells available, the coder did not need to break dwells when, e.g., blinks occurred. For OpenFace estimates, the AOI boundaries were first defined based on the spatial distributions of the horizontal gaze data. For estimates close to the borders of two AOIs, even small variability could potentially break a dwell. As demonstrated in the results of the first experiment, in many cases the accuracy and precision of OpenFace was not sufficient to distinguish between fixations on the nine stimulus points displayed on a screen. The consistently shorter mean dwell durations seen in the results of this experiment suggest that the data quality of OpenFace gaze estimates concerning infant participants is too low to reliably determine the starts and ends of dwells even in situations with sparse, horizontally separated AOIs.

### Interim summary

In the second experiment, we showed that when looking at the relative total dwell time to the AOIs for the whole group of infants, similar conclusions could be made using OpenFace gaze estimates and manual coding. When examining the results on the individual level, OpenFace-based relative total dwell time was on average a reliable measure for the AOIs that were looked at the most (the “left lamp” and “right lamp” AOIs), but less so for the AOIs looked at least (the “parent” and the “none” AOIs). OpenFace-based mean dwell duration, on the other hand, was highly unreliable for all the AOIs.

## General discussion

For computer-vision-based gaze estimation to see widespread use in psychological research, the average researcher needs to know, at a minimum, (1) which methods can be used and (2) how they perform within the contexts of their experimental setups. In the following sections, we address both points.

### Which methods are usable for the experimental psychologist?

We consider a method to be usable by the experimental psychologist if there exists sufficient documentation for download, installation, and use of the method. In essence, this means that the available documentation should allow the user to go from the point of not having anything installed to the point where it is possible to feed a video to the method and read and understand the gaze prediction output it provides. From the long list of potential computer-vision-based gaze estimation methods presented in the introduction, just this requirement already narrowed the list down to only two toolkits: OpenFace and OpenGaze. As it currently stands, the number of methods available to the experimental psychologist is highly limited.

#### The usability of OpenFace

OpenFace, a software toolkit employing a model-based gaze estimation approach, is relatively straightforward to install and use. There are no specific hardware dependencies, only a few software dependencies, and the wiki (found at https://github.com/TadasBaltrusaitis/OpenFace/wiki) provides step-by-step instructions for downloading and use of the toolkit. The output format for gaze estimation is explained, although the origin of the coordinate system appears to be omitted. Based on their explanation of how gaze direction is computed, we assume the origin to be the camera (see Baltrušaitis et al., 2018). The experimental psychologist interested in applying computer-vision-based gaze estimation can thus expect to be able to install and use the OpenFace toolkit for gaze estimation relatively easily.

#### The usability of OpenGaze

The usability of OpenGaze is a different story. OpenGaze is claimed by the developers to be “the first software toolkit for appearance-based gaze estimation and interaction” (Zhang et al., 2019, p. 1). The OpenGaze wiki (found at https://git.hcics.simtech.uni-stuttgart.de/public-projects/opengaze/wiki) provides step-by-step instructions on how to install OpenGaze. The developers have successfully tested and used OpenGaze on Ubuntu Linux 16.04 LTS. Multiple software dependencies are listed. The software had been tested on specific versions of the NVIDIA CUDA Toolkit, the NVIDIA CUDA Deep Neural Network library (cuDNN), and the deep learning framework Caffe. Installation, even when following the step-by-step instructions, was an arduous process that required us to test various graphics processing units (GPUs), different versions of CUDA and cuDNN, compiling dependencies such as Caffe, OpenCV, and Dlib from source, until we finally arrived at a working installation. This was only achieved using an older generation NVIDIA GeForce GTX 1080 Ti GPU, which is now largely unavailable. In sum, downloading and installing OpenGaze was possible, but required an extensive period of trial and error, specific hardware, previous experience using Linux, and dealing with various problems related to compiling programs from source and resolving errors. One might argue that these are not skills an experimental psychologist typically possesses.

Interpreting the data provided by OpenGaze was also problematic. The OpenGaze wiki states that a 3D gaze direction vector should be included in the output. This would have been desirable, as 3D gaze direction vectors could have been transformed into gaze angles and our aim was to examine how the gaze predictions of the method related to the world. Using OpenGaze, we were able to produce an output text file that included 14 columns of comma-separated values. This text file, however, did not include any labels for those values. In fact, to make sense of any of the provided data, we had to examine the OpenGaze code to know what data each column represented. In the output files, we could not find any indication of a 3D gaze direction vector. Instead, we could only find 2D gaze estimates in normalized coordinates. Normalized coordinates represent positions on an assumed screen of a specified size. To relate them to the world, however, one needs to know where in the world the assumed screen is and what its dimensions are. The wiki page states that a custom configuration file for the camera and screen can be used and points to the files containing the default values. However, the values within the files are given and entered in a form that is unusable by someone without the required expertise in computer science, and no documentation on how to understand or change them is provided. Thus, we were able to produce gaze estimates but could not directly relate them to the world. The usability of OpenGaze in terms of interpreting the output was highly lacking. We emailed the developers regarding the problems we faced but never received a response. These issues, as well as the ones we faced when trying to get Gaze360 (Kellnhofer et al., 2019) to run, reflect the fact that the developers of these methods have a different target group in mind than applied researchers. It seems that the field is advancing at such a pace that even the projects that do put effort into making their methods available quickly fall through and become forgotten.

This further brings us to the reasons why we could not use OpenGaze to process the recordings of the second experiment. Our main problem was that the OpenGaze setup we had with great difficulty managed to get to work was at Utrecht University (the Netherlands), while the infant data for the second experiment was stored at Uppsala University (Sweden). We could either (1) transfer the video data from Uppsala to Utrecht, (2) ship the OpenGaze computer to Uppsala, or (3) install OpenGaze on a separate computer in Uppsala. Given the issues concerning the usability of the software, not wanting to risk our only working setup, and not having permission for data sharing of infant videos, none of these options was feasible. These problems illustrate the importance of a well-maintained package that is easy to install and use, of which the OpenFace toolkit is a great example. Collaborative research projects often require one to process data in different locations. If the methods are difficult to install, researchers lacking the required expertise will not be able to make use of them.

### What to expect from the methods in terms of performance?

The first experiment was designed to evaluate the gaze prediction performance of OpenFace and OpenGaze under optimal conditions. With the results, we showed that the gaze predictions by OpenGaze can potentially be used to make distinctions between gaze to stimuli separated by at least 11 degrees with respect to the participant’s eyes, with both horizontally and vertically separated stimuli at a distance of 85 and 95 cm from the camera and screen, respectively. When concerned with stimuli separated by 7 degrees in the vertical direction, the method did not perform as well. These results are in line with Zhang et al. (2019), who showed that when uncalibrated and viewing at a distance of 75 cm from the camera, the gaze estimation method in OpenGaze produced a mean error of 6.4 degrees. Importantly, Zhang et al. (2019) also showed that mean gaze estimation error was largely invariant to changes in viewing distance (tested at 30, 50, 75, 110, 140, and 180 cm) and remained stable across different settings (i.e., indoors vs. outdoors), although these results were based on gaze predictions involving a person-specific calibration of 60 samples.

Contrarily, the gaze estimation performance of OpenFace was highly variable. Its estimates were not reliable enough to make distinctions between gaze to stimuli separated by 11 degrees of gaze angle. This is further corroborated by results showing that even the state-of-the-art model-based gaze estimation algorithm GazeML (Park et al., 2018), which reportedly performs better in terms of mean error than the algorithm OpenFace uses, had an error of 12.1 degrees when operating uncalibrated and at a distance of 75 cm (Zhang et al., 2019). Researchers looking to use OpenFace should take care when drawing conclusions based on OpenFace estimates, as they can vary greatly depending on whether one fixates with only the eyes or with the eyes and head together. We conclude that under optimal conditions and at the distances and horizontal stimulus angles of our setup (see Fig. 1), OpenGaze is viable but OpenFace is not.

In the second experiment, we compared the gaze estimation performance of OpenFace to gaze estimates manually coded directly from the video. A previous study by Eschman et al. (2022) has shown that when coupled with further processing using an artificial neural network trained on OpenFace data with infants, high agreement between automatic and manual coding can be obtained (to note, in addition to gaze estimates, landmark data, head pose data, shape parameters, and facial action units were further utilized to train the network). The researchers collected video recordings of faces of infants and toddlers of different ages both in the lab and remotely. Their results showed high agreement between automatic and manual coding for both the offline (agreement rates of 89.9% and 85.83% for 6- and 36-month-old infants, respectively) and online conditions (mean agreement rate of 90.7% for 48-, 60-, and 72-month-old infants), indicating that with young children and under specific conditions (i.e., three horizontally separated AOIs and further utilizing an artificial neural network), OpenFace can potentially be used to reliably derive relevant gaze measures. In our study, we did not employ further processing with a neural network. Rather, we directly examined dwell-based gaze measures based on OpenFace estimates and manual coding. We showed that OpenFace-based relative total dwell time was highly similar to that obtained by manual coding for looks to the major attention grabbers (i.e., the left and right lamp). However, when determining when infants looked at the face of their parent and when determining which looks did not belong to any AOI, the results were less reliable. This can potentially be explained by how infants looked at the AOIs. When manually coding gaze direction from videos, we noticed that infants often looked at a lamp and then back at the face of their parent using only the eyes while maintaining the head oriented toward the lamp. When looking away, they often displayed similar behavior, such as looking toward the ceiling with only the eyes while the head was oriented forward. As we have shown, OpenFace produces different estimates when looking with only the eyes compared to when looking with both the head and the eyes. It may be that OpenFace had the most problems with this type of gaze behavior. Furthermore, measures for mean dwell duration based on OpenFace estimates were found to be highly unreliable for all the AOIs. It appears that when dealing with uncalibrated OpenFace output and with infant participants at the distances specified in our setup (Fig. 7), relative total dwell time to sparse horizontally separated AOIs is somewhat reliable, but measures examining dwells more closely, such as mean dwell duration or total number of dwells, are not. Fewer AOIs, more separation between AOIs, closer camera placement, or different methods for defining AOIs may lead to better outcomes.

### Advantages, limitations, and future considerations

As we see it, one major advantage of computer-vision-based gaze estimation is the freedom in positioning of the recording equipment. It is often easier to position a camera than, for example, a remote eye tracker, which requires one to make sure that it is positioned correctly in front of the participant and that the head is within a predefined area (to note, some remote eye trackers allow for more freedom in positioning than others but may require custom illumination setups). Cameras can be equipped with various lenses, positioned further away and at different angles, and can be used under normal room lighting. Moreover, as Zhang et al. (2019) have shown, appearance-based approaches can potentially be highly stable across various distances and settings. Whether filming the face from a different angle leads to a difference in the gaze evaluation performance of computer-vision-based gaze estimation is also important to assess in future evaluations. Guidelines for required camera positioning to distinguish between specific AOI configurations could be highly useful for researchers interested in the methods. A second important advantage is the absence of a mandatory calibration. Many commercial eye trackers need to first be calibrated with the person being recorded. Experimental situations where time is of concern (e.g., when dealing with infant participants or multiple recordings in quick succession) or calibration is difficult (e.g., with infant participants) could greatly benefit from not having to calibrate before each recording.

The major disadvantages of computer-vison-based gaze estimation relate to the usability of the methods. First, as we have shown, only two methods made it through our relatively liberal criteria. For computer-vision-based gaze estimation to become available for a wider audience, the developers of these methods need to publish them for more general purposes. Second, there must be clear instructions on how to interpret the output data. Currently, this is not done sufficiently even for the OpenFace toolkit, as the origin of the coordinate system is not clearly stated. For most use cases in experimental psychology and related fields, the main concern of researchers is when and for how long one looks at a particular AOI. If the methods do not allow the user to relate the output data directly to the world, examining looks to AOIs is not a straightforward process and requires one to make further assumptions (e.g., assuming a cluster of gaze samples to represent looks to the same AOI).

Another notable disadvantage is the gaze estimation performance of the available methods. We have shown that when operating uncalibrated, OpenGaze can be used reliably under optimal conditions in situations where stimuli are separated by at least 11 degrees, while OpenFace cannot. When considering research questions such as where on the face of another person one looks during face-to-face interaction, the distances between relevant AOIs tend to be much smaller. If computer-vision-based gaze estimation is to be applied in such situations, the data quality of the estimates they produce needs to improve.

With the recent increased awareness and development of using AI to solve complicated tasks in user-friendly ways (i.e., ChatGPT, Bing, etc.), initiatives such as OpenFace and OpenGaze may be improved or bypassed by similar efforts in the near future. In light of this, we emphasize that computer-vision-based gaze estimation could be made more accessible. We believe that widespread use is the most efficient way to make progress within the field.

## Conclusion

Computer-vision-based gaze estimation can potentially be used in screen-based experiments under optimal conditions with adult participants as well as in experiments with more sparsely separated stimuli and infant participants. With the results of our two experiments, we have shown that to reliably employ the available computer-vision-based gaze estimation methods, one needs to pay close attention to which methods work in which situations and for which types of measures. Moreover, as it currently stands, the lack of documentation and number of methods usable by the average researcher without experience in computer science is far too limited. For the methods to see more widespread use and further validation in the fields of, e.g., psychology and education, we stress the importance of providing proper documentation for installation, use, and interpretation of the data. We hope that future researchers and developers can make use of our results and suggestions.
